# Early *Lotus japonicus* root transcriptomic responses to symbiotic and pathogenic fungal exudates

**DOI:** 10.3389/fpls.2015.00480

**Published:** 2015-06-29

**Authors:** Marco Giovannetti, Alfredo Mari, Mara Novero, Paola Bonfante

**Affiliations:** ^1^Department of Life Science and Systems Biology, Università degli Studi di TorinoTorino, Italy; ^2^Scuola Superiore Sant’Anna di Studi Universitari e PerfezionamentoPisa, Italy

**Keywords:** presymbiotic phase, defense response, microarray, *Lotus japonicus*, germinating spore exudates, chitin oligomers, arbuscular mycorrhizal symbiosis

## Abstract

The objective of this study is to evaluate *Lotus japonicus* transcriptomic responses to arbuscular mycorrhizal (AM) germinated spore exudates (GSEs), responsible for activating nuclear Ca^2+^ spiking in plant root epidermis. A microarray experiment was performed comparing gene expression in Lotus rootlets treated with GSE or water after 24 and 48 h. The transcriptional pattern of selected genes that resulted to be regulated in the array was further evaluated upon different treatments and timings. In particular, Lotus rootlets were treated with: GSE from the pathogenic fungus *Colletotrichum trifolii*; short chitin oligomers (COs; acknowledged AM fungal signals) and long COs (as activators of pathogenic responses). This experimental set up has revealed that AM GSE generates a strong transcriptomic response in Lotus roots with an extensive defense-related response after 24 h and a subsequent down-regulation after 48 h. A similar subset of defense-related genes resulted to be up-regulated also upon treatment with *C. trifolii* GSE, although with an opposite trend. Surprisingly, long COs activated both defense-like and symbiosis-related genes. Among the genes regulated in the microarray, promoter-GUS assay showed that LjMATE1 activates in epidermal cells and root hairs.

## Introduction

Since the land conquest about 450 million years ago, plants had to deal and relate with both pathogenic and beneficial organisms. On the one hand, plants had to protect themselves from pathogens, evolving strong defense mechanisms to effectively ward them off (Jones and Dangl, 2006). On the other hand, plants have developed symbiotic relationships based on a fair nutrient exchange (Kiers et al., 2011). Understanding how plants can discriminate between friends and foes is a crucial question in plant biology (Hayashi and Parniske, 2014; Bonfante and Genre, 2015) with a direct effect on agricultural practices. On the pathogen side, a big effort has been invested in the study of fungal and bacterial effectors and plant receptors, focusing on the modulation of plant immunity, plant disease resistance and its application in modern agriculture (Dodds and Rathjen, 2010; Macho and Zipfel, 2014).

Among the beneficial microorganisms capable to form symbiosis with plants, research has mainly been guided by nutritional aspects (Gutjahr and Parniske, 2013) with less focus on immunity and compatibility aspects (Rey and Schornack, 2013). The two most studied symbioses between plants and soil microorganisms are symbiotic nitrogen fixation (Gage, 2004) and arbuscular mycorrhizal (AM; Parniske, 2008). Plant genetics and mutant analyses allowed to characterize several genes required for both root endosymbioses (*in primis LjSYMRK*/*DMI2, LjPOLLUX/DMI1, LjCCaMK/DMI3* in the case of the model legumes *Lotus japonicus* and *Medicago truncatula*, respectively), thus defining a CSSP (Oldroyd, 2013).

Knowledge on AM signals perception is not yet fully understood: LCOs have been identified in AM roots and in germinating AM spore exudates using the same bioassay that was set up for Nod factor characterization and named Myc-LCO (Maillet et al., 2011). Myc-LCO can induce lateral root formation, calcium spiking and a huge set of gene regulation but all of these actions are dependent on the MtNFP, the Nod Factor receptor (Maillet et al., 2011; Czaja et al., 2012; Genre et al., 2013). By contrast, it has been demonstrated that tetra- and pentamers of N-acetylglucosamine (chitin oligosaccharides: CO4, CO5), contained in spore GSE, activate the CSSP independently of MtNFP (Chabaud et al., 2011; Genre et al., 2013). Moreover CO4 and CO5 concentration is strongly increased when germinating spores are treated with GR24, a synthetic strigolactone analog. The same molecules have been shown to be active also in rice (Sun et al., 2015) and carrot, but not in *Arabidopsis* (Genre et al., 2013).

Plant–microbe interactions utilize similar chemical signatures to mediate biological processes leading to a symbiotic or a pathogenic relationship (Liang et al., 2013; Evangelisti et al., 2014; Miyata et al., 2014; Wang et al., 2014; Zhang et al., 2015). For example, a cross-talk between the CSSP and chitin-defense responses has been suggested: MtNFP, involved in the formation of endosymbioses, has been shown to be unsuspectedly involved in plant immunity, thus *mtnfp* plants resulted to be more susceptible to pathogens, such as *Aphanomyces euteiches* and *Colletotrichum trifolii*, and its overexpression increased resistance (Gough and Jacquet, 2013; Rey et al., 2013). Another hint about the existence of parallels between Nod factor-induced and chitin-induced signaling, mediated by the respective LysM RLK, is given by the similarity of *Nicotiana benthamiana* responses to MtNFP and MtLYK3 (both part of the Nod factor receptor complex) co-production and AtCERK1 production (Pietraszewska-Bogiel et al., 2013). It was recently shown that OsCERK1, known to be responsible of the detection of pathogenic chitin molecules in rice, is also involved in the interaction with AM fungi (Miyata et al., 2014; Zhang et al., 2015).

Both the Nod Factor receptor and *AtCerk1* and, probably, the still unknown Myc-LCO receptor(s), bind to chitin residues, but the presence of different receptor complexes seems to allow a correct recognition of different chitin molecules and a discrimination between symbiont and pathogen chitin signals (Antolín-Llovera et al., 2014; Cao et al., 2014; Ried et al., 2014).

Fully colonized and functional AM roots were extensively studied by expression profiling, initially with a whole organ approach (Fiorilli et al., 2009; Guether et al., 2009), then through cell-type specific microarray (Hogekamp et al., 2011; Gaude et al., 2012), and more recently with RNA-seq approaches (Ruzicka et al., 2013; Handa et al., 2015). But up to date, genome-wide studies analyzing the presymbiotic stages only considered the transcriptomic impact of Myc-LCOs (Czaja et al., 2012) or the stage of hyphopodium formation (Hogekamp and Küster, 2013). The goal of this investigation is to characterize the transcriptome of *L. japonicus* upon perception of *Gigaspora margarita* GSE. GSE may contain not only a mix of simple sulfated and non-sulfated LCOs (referred to as sMyc- and nsMyc-LCOs; Maillet et al., 2011), COs (Genre et al., 2013), and effectors (Kloppholz et al., 2011; Tisserant et al., 2013) – each one probably playing a role during AM presymbiotic phase (Sun et al., 2015) – but also – still unknown – fungal molecules perceived by plants. This exudate could represent an ideal mix to investigate plant responses to AM fungi, since it better mimics the bioactive molecules released by AM fungi in natural conditions during plant–fungal presymbiotic interaction and defense-like responses. As a second goal, we wanted to go deeper in the characterization of plant-defense genes – which are known to be activated by AM fungi not only in roots but also in other organs like shoots and fruits (Fiorilli et al., 2009; Zouari et al., 2014) – in order to understand whether AM exudates may simultaneously activate both symbiotic and pathogenic-like responses. To validate this hypothesis, we treated Lotus seedlings with short (CO5) and long (CO8) COs, since the first elicit the symbiotic calcium spiking (Genre et al., 2013), while CO8 are the chitooligosaccharides which act as elicitors of defense (Hayafune et al., 2014). In parallel, we tested the specificity of a subset of genes by treating Lotus rootlets with GSE from a pathogen fungus such as *C. trifolii*.

Our genome-wide expression analysis revealed: (i) more than 100 genes induced by the perception of fungal GSE; (ii) a wide and extensive defense-like response in Lotus root 24 h after the perception, and a subsequent down-regulation of defense-like genes after 48 h, (iii) the activation by CO8 of both defense-like and symbiosis-like genes, (iv) similarities between the symbiotic and pathogenic signature elicited by the AM and *C. trifolii* GSE, and (v) the localization of one of the activated genes in epidermal cells by means of promoter-GUS assays.

## Results

### *G. margarita* GSE Triggers a Specific Gene Expression

**FIGURE 1:**
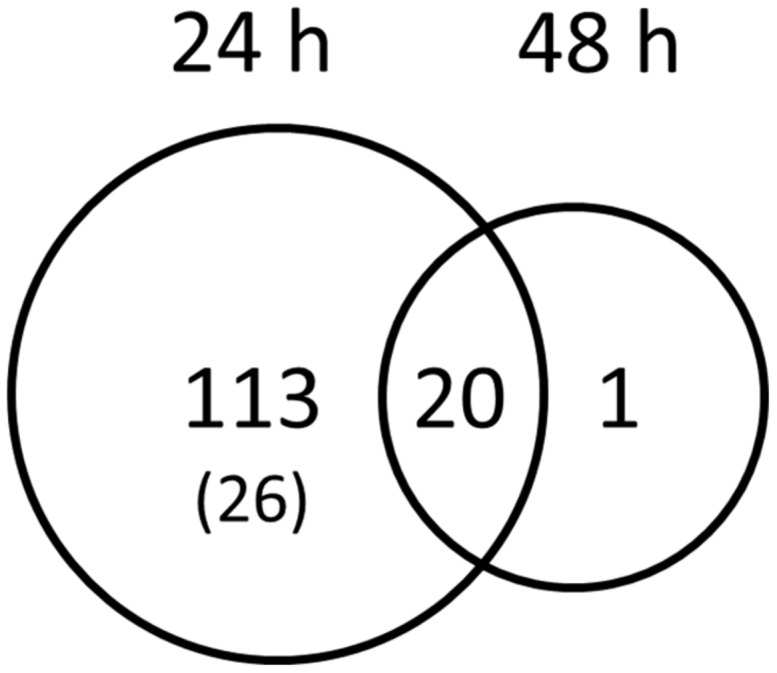
**Genes specifically activated by *Gigaspora margarita* spore germination water.** The Venn diagram visualize the coactivation of *Lotus japonicus* wild-type root genes by GSE 24 and 48 h after treatment. Numbers indicate the number of genes with FDR values <0.05 and a fold change cut-off < -1 or > +1. The number into brackets indicate the genes that resulted to be down-regulated.

To record transcriptional responses toward symbiotic signals, we treated *L. japonicus* wild-type roots with *G. margarita* GSE. A microarray experiment with RNA coming from Lotus rootlets treated with GSE allowed us identifying 134 genes differently regulated after 24 h of treatment with GSE and 21 genes after 48 h (**Figure 1**). One third of the genes resulted to be linked to defense or redox mechanisms: they showed an up-regulation after 24 h and most of them had a dramatic down-regulation after 48 h (**Table 1**), probably pointing to a defense-like response of the plant to the AM fungal exudates and a subsequent down-regulation of that response (data sheet 1).

**Table 1:** List of gene categories regulated in the microarray experiment.

Putative function	# of genes
Redox related genes	35
Defense/stress related genes	19
Hormone related genes	7
Transporters	6
Signaling related genes	5
Secondary metabolism	8
Other functions	54
	134

Among the most up-regulated genes at 24 h, **Table 2** shows a pathogenesis-related protein, a lipase-hydrolase protein and various glutathione-*S*-transferases (GSTs), revealing a response of the root to microbe signals in line with responses already registered in plant root hairs upon early infection by symbiotic soil bacteria (Libault et al., 2010; Gourion et al., 2015). Among the most down-regulated genes, we identified genes linked to the phospholipid metabolism, such as a putative phosphatidylinositol phosphatidylcholine transfer protein, and to the ion traffic.

**Table 2:** Most up- and down- regulated genes in *Lotus japonicus* wild-type roots treated with *Gigaspora margarita* GSE.

Lotus GeneChip ID	Annotation	Log2 FC		*p*-values	
		24 h	48 h	24 h	48 h
Ljwgs_075692.1.1_at	Lipase hydrolase-like protein	4.92	–0.52	0.000	0.369
Ljwgs_079986.1_s_at	PR10-1 protein	4.82	0.73	0.000	0.205
Ljwgs_044810.1_at	Bark leucoagglutinin I precursor	4.57	0.77	0.000	0.334
TM1656.16_at	Pectinesterase	4.22	–0.17	0.000	0.808
Ljwgs_051780.1_at	UDP-glucose:SA glucosyltransferase	3.96	4.60	0.000	0.000
chr1.CM0064.61_s_at	Germin-like protein	3.95	2.23	0.000	0.006
Ljwgs_028218.2_at	Glutathione S-transferase GST 9	3.90	3.50	0.000	0.000
Ljwgs_075865.1_at	Bark agglutinin I, polypeptide B precursor	3.77	0.83	0.000	0.200
chr6.CM0437.7_at	MTN19 gene precursor	3.21	1.56	0.000	0.016
chr5.CM0909.45_at	Glutathione-*S*-transferase GST 15	3.15	3.36	0.000	0.000
chr3.CM0208.34_at	Putative Fe(II) ascorbate oxidase	3.10	0.12	0.000	0.740
chr2.CM0201.55_at	Auxin-induced protein	3.08	3.96	0.000	0.000
chr4.CM0046.42_at	Glutathione-*S*-transferase GST 14	3.02	2.36	0.000	0.000
chr1.CM0010.42_at	Purple acid phosphatase (PAP22)	–1.52	–0.25	0.000	0.300
Ljwgs_066244.1_at	Putative glucanase	–1.74	–0.10	0.000	0.689
chr4.CM0126.67_at	Hypothetical protein	–1.80	–0.25	0.000	0.422
Ljwgs_122957.1_at	Cyclic nucleotide and calmodulin-regulated ion channel-like protein	–1.83	–0.24	0.000	0.406
Ljwgs_025743.1_s_at	Cyclic nucleotide and calmodulin-regulated ion channel-like protein	–1.83	–0.22	0.000	0.393
Ljwgs_071032.1_s_at	SEC14 – like protein	–1.88	–0.19	0.000	0.509
chr4.CM0399.52.1_at	AP2 domain containing protein RAP2.11	–1.91	0.01	0.000	0.979
Ljwgs_007118.2_at	Unknown protein	–1.93	–0.25	0.000	0.412
chr5.TM1125.10_at	Glutathione peroxidase -like protein	–2.11	–0.03	0.000	0.925
Ljwgs_071601.1_s_at	Putative phosphatidylinositol phophatidylcholine transfer protein	–2.19	–0.15	0.000	0.587

*Gene expression is represented by Log2-fold change values for the two treatments (24 and 48 h after GSE application), indicating also the p-values.*

### AM-GSE Activate Genes in an Opposite Way than Pathogen GSE

Looking at the relevant Lotus defense responses elicited by the GSE (one third of the differentially expressed genes were related to defense) we wondered whether this gene subset represented a part of the genes required for the establishment of AM symbiosis or whether it mirrored mostly a defense response, similar to the one elicited by pathogenic fungi.

**FIGURE 2:**
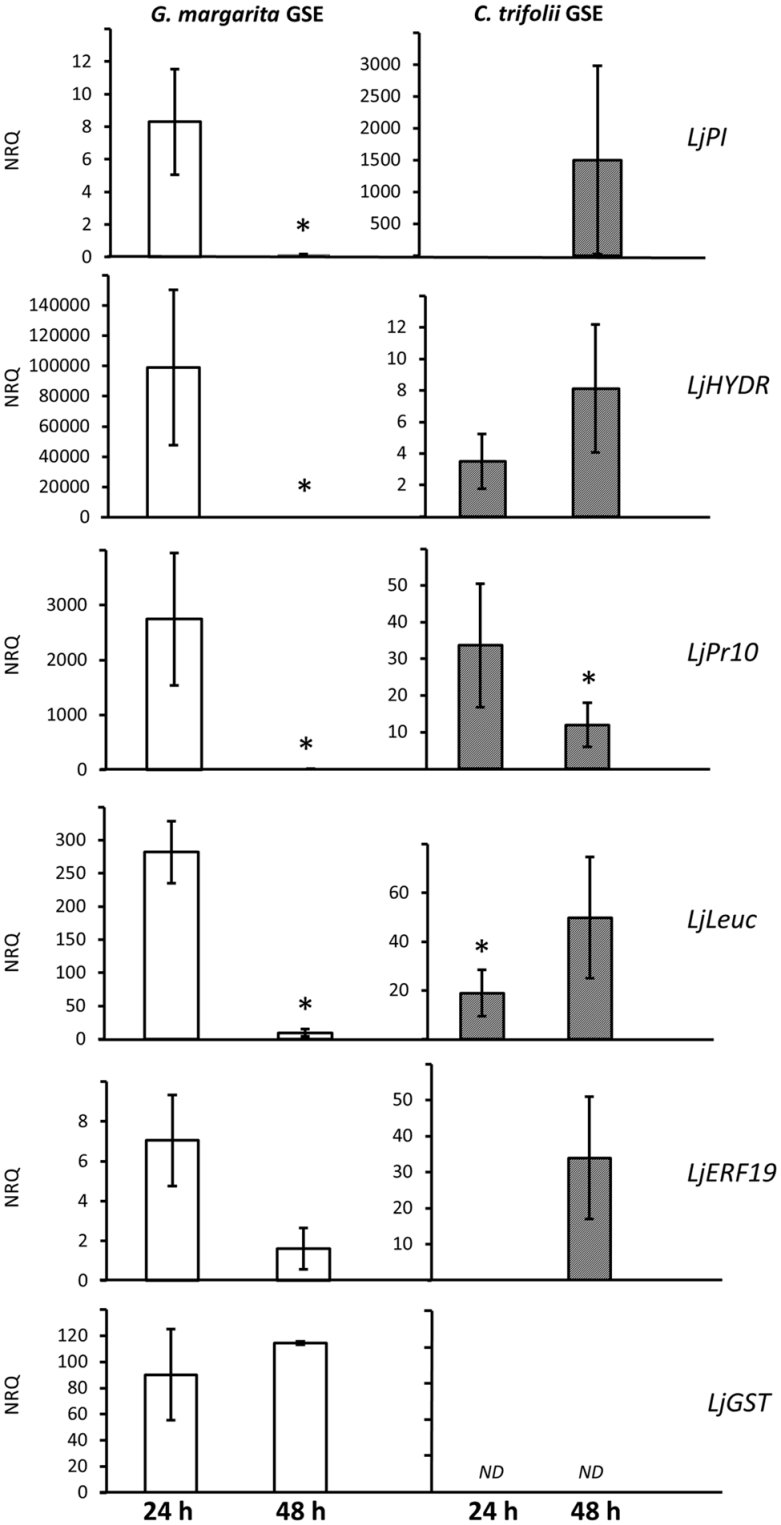
**Comparison of gene induction by *G. margarita* or *C. trifolii* GSE 24 and 48 h after the treatment.** Putative defense-like genes were selected from the microarray list and their gene expression tested in a new experiment with also germination water from a fungal pathogen, *Colletotrichum trifolii*, after 24 and 48 h. Bars represent the mean of four biological replicates ± SE. Bars subtended by the asterisk differ significantly at *p*-value < 0.05, according to the Kruskal–Wallis test. NRQ stands for Normalized Relative Quantities. LjPI, proteinase inhibitor; LjHYDR, hydrolase-like protein; LjPR10, pathogenesis related protein; LjLeuc, Bark leucoagglutinin precursor; LjERF19, ethylene responsive element; LjGST, glutathione-*S*-transferase.

To better understand this point, from the microarray data we selected four genes that were strongly down-regulated between 24 and 48 h, one gene that resulted to be up-regulated at both time points and one putative marker gene (LjERF19). We compared the relative expression of these six genes over two time points (24 and 48 h) with the gene induction generated by spore germination exudates from a fungal pathogen, *C. trifolii*. This biotrophic pathogen is known to trigger early cell responses in the root epidermis (Genre et al., 2009), but its GSE (which contains COs of variable length) cannot activate the CSSP (Genre et al., 2013). **Figure 2** shows that four out of five selected genes (a protein inhibitor-LjPI, a hydrolase-LjHydr, a pathogenesis related protein-LjPR10 and a lectin-like proteins-LjLeuc) when treated with *G. margarita* GSE are highly overexpressed at 24 h and then dramatically down-regulated, confirming the data coming from the microarray. By contrast, the treatment with *C. trifolii* GSE resulted in an opposite behavior, showing a slower increase of expression between 24 and 48 h. The only exception was represented by *LjPR10* that was down-regulated with a similar expression pattern than AM fungal exudates.

Finally we checked for the trend of a LjGST, one of the few genes that in the microarray was overexpressed at both time points. QPCR confirmed this trend with AM GSE treatment but the transcript was not detectable after treatment with pathogenic GSE. LjGST function within AM development is still unknown but the gene is overexpressed also in later stage of symbiosis (Wulf et al., 2003; Hao et al., 2012; Hogekamp and Küster, 2013).

In addition, to understand if GSE could contain not only bio-active molecules involved in signaling, but also effectors down-regulating host defense pathways, we checked for the expression level of *LjERF19*. *LjERF19* is the Lotus ortholog of *MtERF19*, a transcription factor targeted and down-regulated by AM fungal effector SP7 (Kloppholz et al., 2011). As reported for *Medicago*, we show a down-regulation of the transcription factor from 24 to 48 h after AM but not *C. trifolii* GSE treatment. This result can indirectly show the presence of effectors in *G. margarita* GSE, specifically targeting LjERF19.

Taken in the whole, the qRT-PCR experiment suggests that the plant genes regulated by pathogenic or symbiotic signals are largely overlapping with a specific trend of expression. The results therefore suggest a possible role represented by the timing of the gene activation that could be responsible of triggering different physiological plant responses.

### Short and Long Chitin Oligomers Elicit Different Gene Expression

**FIGURE 3:**
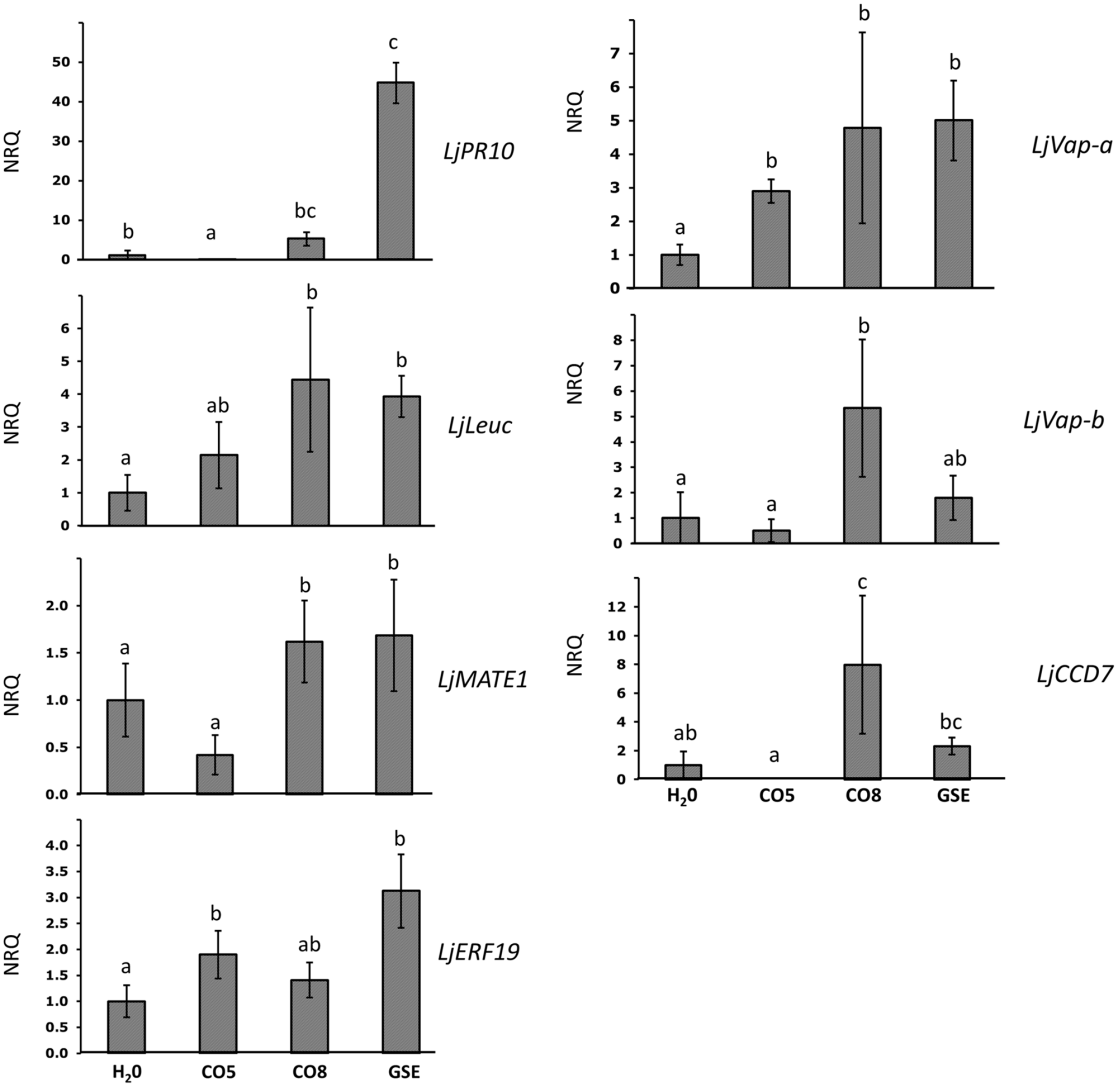
**Relative expression of symbiotic and defense-like genes after treatment with water, short (CO5), long (CO8) chitin oligosaccharides and *G. margarita* GSE, 1 h after the treatment**. The expression of genes previously characterized as related to defense or symbiosis was characterized after treatment of Lotus rootlets with short or long chitin oligosaccharides. As controls we used *G. margarita* GSE and water. Bars represent the mean of four biological replicates ±SE. Bars subtended by the same low-case letter do not differ significantly at *p*-value < 0.05, according to the Kruskal–Wallis test and Mann–Whitney *Post hoc* test. NRQ stands for Normalized Relative Quantities. LjPR10, pathogenesis related protein; LjLeuc, Bark leucoagglutinin precursor; LjMATE1, multidrug and toxic compound extrusion gene; LjERF19, ethylene responsive element 19; LjVap, vapyrin-like gene; LjCCD7, carotenoid cleavage dioxygenase 7.

On the basis of insights on the composition of active and non-active chitin-related oligomers contained in AM and *C. trifolii* exudates (Genre et al., 2013), and our RT-PCR experiments (**Figure 2**), we wondered whether short (CO5) and long (CO8) COs could partly mimic symbiotic and pathogenic signals, respectively. It is known that CO5 are able to induce Ca^2+^ spiking (Genre et al., 2013), while CO8 which miss to elicit the symbiotic-calcium spiking, can be associated to the defense-responses previously identified (Liang et al., 2014). Moreover we hypothesized that *G. margarita* GSE could contain a variety of chitin molecules and therefore able to trigger both symbiosis and pathogenic related genes. To test this, we performed a set of qPCR on Lotus rootlets treated with water, short (CO5), long (CO8) COs, and *G. margarita* GSE focusing on an earlier time point, 1 h after treatment, in order to be closer to the calcium spiking events (**Figure 3**). We chose some defense-related genes activated in the microarray and some other genes that could be considered gene marker of AM development. The treatment inducing the highest regulation of genes was GSE. Consistently with our prediction, defense-like genes, such as *LjPR10, LjLeuc* and *LjMATE1*, resulted also to be induced both by CO8 and GSE but not by water and CO5 treatment. Surprisingly, CO8 were also able to induce genes involved in mycorrhizal colonization, such as vapyrin genes (*LjVap-a* and *LjVap-b;* **Figure 3**), regardless of the fact that the CO8 are not able to elicit a nuclear calcium spiking. At the same time, CO8 can activate a key gene in the metabolic pathway of strigolactone synthesis (*LjCCD7*). Overall, CO5 were less active than CO8 with the exception of the positive regulation of *LjERF19*, the ortholog of *MtERF19* (Kloppholz et al., 2011).

### Promoter GUS Localization

**FIGURE 4:**
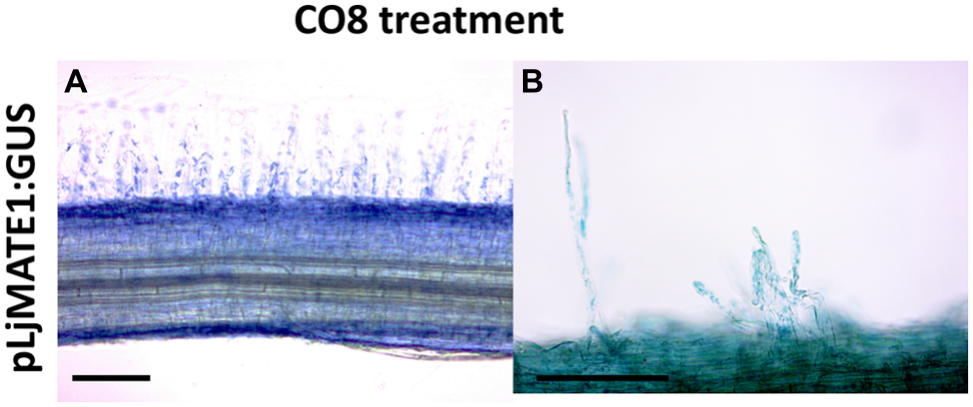
**Histochemical GUS staining of *L. japonicus* roots expressing pLjMATE1:GUS.** After treatment with long COs (CO8), *L. japonicus* roots expressing pLjMATE1:GUS shows a blue coloration that seems to regard exclusively epidermal cells **(A)** as hinted by the typical blue signal coming from root hairs **(B)**. Cortical and central cylinder cells are not marked. Scale bars corresponds to 100 μm.

To localize Lotus transcriptomic responses to GSE, we focused on LjMATE1, a multidrug and toxic compound extrusion protein that resulted to be up-regulated in the microarray experiment (data sheet 1) and is potentially involved in different kind of cellular detoxification. The same gene is involved in citrate transfer within nodules (Takanashi et al., 2013) and upregulated in mycorrhizal roots (Handa et al., 2015) therefore making it a good candidate for a putative function in legume endosymbioses. The putative promoter region of LjMATE1, 2243 bp upstream of the coding region, were fused to the reporter gene GUS. This construct was introduced into Lotus roots by *Agrobacterium rhizogenes*-mediated transformation. Composite plants were grown in plates, transgenic hairy roots were generated and treated after 4 weeks with long COs (CO8) for 1 h. Roots incubated with GUS buffer overnight at 37°C showed a typical epidermal coloration (**Figure 4A**) with homogenous blue color coming from root hairs (detail showed in **Figure 4B**) and outer root cell layers. In conclusion it seems that the localization pattern of *LjMATE1* is consistent with the functionality of a gene hypothetically involved in the presymbiotic fungal-plant interaction.

## Discussion

The combination of untargeted and targeted transcriptomic analyses has allowed us to add some novel information to the still opened question of plant host responses to AM signals during the presymbiotic phases. In addition, the comparison of the effects elicited by exudates released by symbiotic and pathogenic fungi has revealed how the host plant responds activating the same gene subset but with a specific timing.

### *G. margarita* GSE Trigger *L. japonicus* Transcriptomic Responses

The results of our experiment (the use of the whole GSE which contains a cocktail of known and unknown molecules) led to a first conclusion: *G. margarita* GSE-induced gene regulation in Lotus is highly different than gene expression induced in *Medicago* by purified Myc-LCO (Czaja et al., 2012). Among the 134 genes regulated by GSE in Lotus, just two genes show a significant similarity with *Medicago* genes induced by Myc-LCO treatment, a putative GST (Mtr.18369.1.S1_at) and a putative endoglucanase (Mtr.50565.1.S1_at). These few overlaps could be due to the different biological system or to the different activity exerted by the complexity of GSE.

The induction of gene expression resulted to be mostly transient, consistently with the results obtained with Myc-LCOs and differently from gene regulation by purified Nod-LCOs (Czaja et al., 2012). However, we cannot exclude that the transiency of the response is due to instable nature of AM fungal molecules or, alternatively, to the presence in the GSE of enzymes or other molecules able to inactivate or outcompete for fungal molecules, as it happens with *Cladosporium fulvum* LysM effectors (Sanchez-Vallet et al., 2013). This transiency is well indicated by the low number of regulated genes and the overall highest *p*-value at 48 h. Nor the genes encoding components of the CSSP did show a marked expression change toward GSE, or the genes encoding GRAS transcription factors acting downstream of Ca^2+^ signaling, such as NSP1, NSP2, RAM1 (Gobbato et al., 2012), RAM2 (Wang et al., 2012), or DELLA (Floss et al., 2013): however, it is worth to highlight the fact that all of these genes were identified and characterized through forward genetics and mutant phenotyping and their gene regulation during presymbiotic phase was not described elsewhere.

Among the regulated genes, GSTs represents a major group of detoxification enzymes, regulated *in vivo* by ROS. ROS production at the infection site is the earliest response of PAMP-triggered immunity (PTI). Apart from primary effects, such as cell wall strengthening and induction of antimicrobial activity, ROS function as diffusible second messenger, inducing several resistance responses including synthesis of pathogenesis-related proteins (Zurbriggen et al., 2010), which have been recently demonstrated to be relevant also for symbiotic interaction (Gourion et al., 2015). Other species of oxidative burst are demonstrated to be involved in AM symbiosis as nitric oxide accumulation (Calcagno et al., 2012) and in nodules (Marino et al., 2011).

*Lotus japonicus* medicago truncatula nodulin-like 19 (LjMTN19) seems to be a possible key gene in both nodule and AM formation since its expression is induced both during mature AM colonization in Lotus (Guether et al., 2009) and during nodule formation (Moreau et al., 2011; Naya et al., 2014). Its biological role and function is still unknown but it was shown that Mtn19-like gene from pea increases also in pods treated with the insect elicitor Bruchin B (Doss, 2005) and thus it has been proposed to be involved in plant biotic responses. Our findings support this hypothesis also for Lotus.

Overall many of the genes that showed a highest regulation were previously characterized as involved in plant–microbe interaction: in virus-tobacco interaction, salicylic acid (SA) can activate plant resistance and its levels increase systematically following the hypersensitive response. The SA increase in the inoculated leaf coincided with the appearance of a GSA thanks to the higher activity of UDP-glucose: SA glucosyltransferase (Enyedi and Raskin, 1993). The same class of genes resulted to be regulated in our set up but also involved in *Arabidopsis* resistance against *Pseudomonas syringae* (Boachon et al., 2014).

Altogether novel genes activated by AM fungal exudates have been identified and could constitute a target for future analyses.

### A Cocktail of Specific and Non-Specific Responses

Due to the fact that the majority of genes regulated in the microarray seems not to be specific of AM interactions but belonging to generic plant responses to biotic stress, we wanted to verify whether the same genes could be induced upon contact with GSE from *C. trifolii*, a pathogenic fungus. This experiment allowed to show that most of the genes were still activated but with a completely different pattern of expression: a gradual overexpression over time as opposed to a dramatic down-regulation happening after AM fungal GSE treatment (**Figure 2**). It would be challenging to demonstrate that this expression pattern could mirror the action of effectors probably contained in the GSE. The genome sequence of *R. irregularis* has revealed the presence of hundreds of small secreted proteins (Tisserant et al., 2013). The fungus we used is *G. margarita* which is, among *Glomeromycota*, phylogenetically quite far but we expect biological tools necessary to form a symbiosis to be well conserved among AM fungi.

**FIGURE 5:**
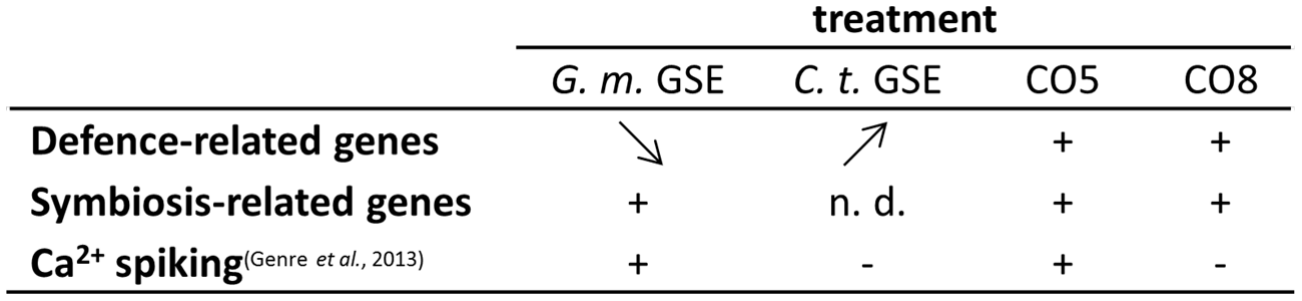
**Activity of fungal exudates and COs.** *G. margarita* GSE (*G. m.* GSE) and *C. trifolii* exudates (*C. t.* GSE) activate a similar set of defense-related genes in *L. japonicus* rootlets, but with a different timing of the expression profile: from high to low (*G. m.* GSE) and from low to high (*C. t.* GSE), as shown by arrow direction. A subset of these genes was shown to be induced also upon treatment with short (CO5) or long (CO8) COs, 1 h after treatment. Symbiosis-related genes were up-regulated by *G. m.* GSE, as expected, but their expression was not discriminated by CO5 and CO8 treatments, differently from calcium spiking activity. This test seems to represent up-to-date the only reliable marker of the presymbiotic phase.

As a further step we wondered whether we could correlate the activity of some chitin oligosaccharides, demonstrated to be present in the GSE, to the different genes activated and, eventually, discriminate between symbiotic and defense-like responses. Genre et al. (2013) showed that CO4 and CO5 are responsible of calcium spiking in transformed roots of *Medicago*, in contrast with longer chitin oligosaccharides such as CO8. Therefore our hypothesis was that long chitin oligosaccharides could be more connected with plant defense response whereas shorter oligomers could be able to trigger mostly genes related to symbiotic pathway or eventually counteract plant immunity system as suggested by (Liang et al., 2014). Comparing our results with results from Czaja et al. (2012) it is worth to highlight the fact that long COs, such as CO8, are sufficient to activate genes essential for the molecular dialog, like the strigolactones (Bonfante and Genre, 2015), as well as genes which allow an efficient epidermal penetration by AM fungi and arbuscule formation, such as Vapyrin (Pumplin et al., 2009; **Figure 3** and **Figure 5**). These results could lead to two different conclusions: AM fungal exudates can contain longer COs with a “symbiotic” function in plant–fungus chemical communication. As an alternative, longer COs, associated with pathogen-like organisms, can activate genes characterized as mycorrhizal specific, as Vapyrin, but with other unknown functions in different plant–microbe interaction, in line with what was shown for *M. truncatula* symbiosis mutants (*mtnfp* and *mtlyk3*, for example) that were affected in the interaction with a biotrophic root pathogen (Rey et al., 2015). To delve into expression pattern of genes regulated in the array and to develop a possible tool and marker of GSE activity, we obtained promoter-GUS lines of one gene of interest. As shown in **Figure 3**, a clear blue coloration confirmed the expression of the gene *LjMATE1* in epidermal cells and root hairs, as we could expect from genes involved in the pre-symbiotic plant–fungal interactions. Promoter-GUS experiment showed that *LjMATE1* is activated in trichoblasts. It is known that Ca^2+^ spiking mainly regards atrichoblasts and AM fungi tend to avoid roots with extensive root hairs, by contrast, it seems that at least one of the genes whose expression is induced upon exposure with fungal exudates, localize at trichoblast level (**Figure 3**), thus providing further evidence of the fact that these response do not overlap with the CSSP. Interestingly LjMATE1 is also involved in nodule maintenance, therefore potentially conserved among the two endosymbioses. In root nodule symbiosis, it was shown to be a citrate transporter and its silencing altered Fe localization in mature nodules of *L. japonicus* (Takanashi et al., 2013; González-Guerrero et al., 2014).

Taken in the whole, our experiments have provided a set of genes which are regulated by the AM signals, have revealed that plant responses are a mix of specific and generic responses and that molecules like CO5, eventhough involved in AM symbiosis, are not strongly involved in transcriptional responses. By contrast, CO8 activate AM symbiotic-related genes, irrespectively, of being traditionally associated to defense-responses. However, irrespectively, of such differences, among the activated genes, LjMATE1 results to be expressed at the plant epidermis providing new tools to investigate the molecular dialog between fungi and their host plants.

## Materials and Methods

### Plant and Fungal Materials

*Lotus japonicus* (Regel) K. Larsen seeds (MG20, WT) were scarified and surface-sterilized for 5 min in concentrated sulfuric acid and washed three times with sterile water. In a second step the seeds were incubated for 3 min in 1:3 diluted commercial bleach with 1:1000 Triton-X. After washing three times with sterile water, the seeds were germinated on water-agar (0.6%) in Petri dishes. For the microarray experiment, three seedlings were left in a 1.5 mL eppendorf tube containing 1 mL of 10X GSE (Genre et al., 2013) or water for 24 and 48 h. *C. trifolii* race 2 strain MUT 3930 (Richard O’Connell, BIOGER-CPP, 78850 Thiverval-Grignon, France) conidia were produced after 7 days at 23°C on a modified Mathur’s medium (0.1% yeast extract; 0.1% BactoPeptone; 1% sucrose; 0.25% MgSO_4_ 7H_2_O; 0.27% KH_2_PO_4_; 2% agar in 1 l of sterile distilled water). Spores were prepared as previously described (Torregrosa et al., 2004). A total of 10^7^ spores were diluted in 100 ml of sterile H_2_O. After 24 h incubation at 24°C, the germinated spores were pelleted by centrifugation at 5000 g for 15 min and the GSE was recovered for analysis. *C. trifolii* GSEs were lyophilized and suspended in 1 mL of H_2_O.

### RNA Isolation and Microarray Hybridization

Roots were harvested and immediately frozen in liquid nitrogen in a 2 ml reaction tube. Two clean metal balls were added into every tube and frozen again. Plant material was then ground using a Retsch^®^ ball mill for 2 min, at least three biological replicate per each condition. RNA was extracted using a modified ‘pine-tree-method’ (Guether et al., 2009). Integrity of RNA samples was checked using an Agilent 2100 Bioanalyzer. RNA purity was determined by ensuring spectrophotometric ratios of A260nm/A280nm ~ 2 and A260nm/A230nm ≥ 2. Removal of genomic DNA was done using the Turbo DNA-free^TM^ reagent (Ambion, Austin, TX, USA) following the manufacturer’s instructions. Absence of genomic DNA was verified by RT-PCR with intron-specific primer for tubulin β-5 (TM0371b.4/TC18284). For each sample, 1 μg of total RNA was send to AtlasBiolabs^1^ to perform the microarray experiment. cRNA was hybridized to the Genechip^®^ Lotus1a520343 and scanned, according to the manufacturer’s instructions.

### Data Analysis

Microarray data were analyzed using the bioconductor software package for the R programming language (Gentleman et al., 2004). Data quality was assessed using the AffyPLM packages (Gautier et al., 2004), and expression estimates were obtained using the RMA algorithm (Irizarry et al., 2003). Control and bacterial probe-sets were removed, and only genes assigned as present (*P* < 0.05) using the MAS5 present/absent algorithm were retained. Statistical testing for differential expression was performed using mixed models with the LIMMA bioconductor package (Smyth, 2004). Comparison of the obtained data sets to the previous published microarray studies were based on TBLASTX. For the *L. japonicus* data sets (Deguchi et al., 2007) an e-value threshold of 1e–50 was applied. Furthermore, microarray data are available in the ArrayExpress database^2^ under accession number E-MTAB-3119.

### cDNA Synthesis and Real Time RT-PCR

Real-time experiments were carried out on material derived from root. cDNA synthesis was performed using SuperScriptII^®^ Reverse Transcriptase and 1 μg of total RNA, following the protocol of the supplier (Invitrogen Ltd, Paisley, UK). Oligonucleotide sequences of all the primers are listed in **Table 2**. Quantitative RT-PCR was carried out with an iCycler apparatus (Bio-Rad Laboratories, Hercules, CA, USA). Each PCR reaction was carried out in a total volume of 15 μl. The following PCR program was used: 95°C for 90 s, 40 cycles of 95°C for 15 s, 60°C for 30 s. A melting curve (54–70 steps with a heating rate of 0.5°C per 10 s and a continuous fluorescence measurement) was recorded at the end of every run to exclude primers generating non-specific PCR products (Ririe et al., 1997). All reactions were performed for at least three biological and two technical replicates. Baseline range and CT values were automatically calculated using the iCycler software. In order to compare data from different PCR runs or cDNA samples, CT values of all genes were normalized to the CT value of UBQ10 (chr1.TM0487.4) as previously described (Guether et al., 2009). **Table 3** indicates the list of primers used in this experiment. Handling small sample size, statistical differences for QPCR analysis were calculated by Kruskal–Wallis non-parametric one-way ANOVA followed by Mann–Whitney *Post hoc* test.

**Table 3:** List of primers used in this study.

Gene locus	Gene name	Primer forward	Primer reverse
Ljwgs_079986.1	LjPR10	CTAAAGGTGATGCTAAACCC	GCAAGCACTTAGAAAGAAGC
Ljwgs_044810.1	LjLeuc	CCAGAGTTTGTCAGAGTTGG	GACTTAGCTAATCAAGTCCG
chr2.CM0201.55	LjQOX	GCAACCTTACTTAGAAAGTGG	CAATTGATTATGCTCATGGG
chr5.CM0909.44	LjGST	GCATTTCTATCGTTTAGAACC	ACATCAAGAAGACAAACCCA
Ljwgs_075692.1.1	LjHydr	GGAATTCACACAACAGAAGC	CATAAAATGAATGGTCATGC
TC11891	LjPI	GAAATGTGCAAGAAATTTCC	AATAAAAGTCTCGCTATCTCC
B3IX38	LjERF19	TAGAGCCTACGATCGAGAAG	AACGACGAGTTTGAACAGAG
chr1.CM1409.130.r2.d	LjMATE1 for	GGGGACAAGTTTGTACAAAAAAGC	GGGGACCACTTTGTACAAGAAAGCT
	GUS vector	AGGCTTGTCACGTCTGACTCCTGAA	GGGTTTTCAATTGTTTGCCTTGTTGT
chr1.CM1409.130.r2.d	LjMATE1	GTGACAGTGCTTACATCG	TAACAAAGGTGACAAATCC
GU441766	LjCCD7	GTATGGAGTGTTTAAGATGCCC	TAAAATGACTGCGTGGAAGC
TC14054	LjUBI	TTCACCTTGTGCTCCGTCTTC	AACAACAGAACACACAGACAATCC
chr1.CM0104.3050.r2.a	LjVAP.a	GCTATCTCACAGAAGAGACC	AACAGAGTCACCAGAACC
LjSGA_008026.1	LjVAP.b	CATGTAGAGGTTCTGAGG	CTGTATCACCTTCTCTGG

### Biochemical Used for Bioassays

Short-chain COs were purchased from Yaizu Suisankagaku Inudstry Corporation (Tokio, Japan). CO8 was kindly provided by Dr. Naoto Shibuya from Meiji University, Kawasaki, Japan to Andrea Genre. The preparation of solutions was performed following protocols indicated by Genre et al. (2013).

### Histochemical Analysis of Root Tissue

Two thousand two hundred forty three base pairs upstream of LjMATE1 cds, one of the genes up-regulated in the microarray, was fused with the GUS gene in the vector pKGWFS70 in order to verify its activation at root level. The red fluorescent marker DsRED, under the control of the constitutive *Arabidopsis* Ubiquitin10 promoter (P*Ubq10*; Limpens et al., 2005), was inserted. *L. japonicus* composite plants carrying transformed roots were treated with water or long chitooligosaccharides. Root fragments, showing DsRED fluorescence were selected under a stereomicroscope. After the treatment, the root segments were covered with freshly prepared GUS buffer [0.1 mM sodium phosphate buffer, pH 7, 0.5 mm K_4_Fe(CN)_6_, 5 mM K_3_Fe(CN)_6_, 0.3% Triton X, 0.3% X–Glc]. Samples were incubated at 37°C for 16 h in the dark, washed with distilled water and observed under an optical microscope (Eclipse E400; Nikon).

## Author Contributions

MG participated in the design of the experiment, carried out most of the experimental part and drafted the manuscript. AM produced exudates from *C. trifolii* and perform part of the qPCR and the GUS assay; MN followed the plant cultures, the spore germinating exudates production and the sampling for the microarray experiment; PB conceived the study, participated in its design and coordination and wrote the manuscript. All authors read and approved the final manuscript.

## Conflict of Interest Statement

The authors declare that the research was conducted in the absence of any commercial or financial relationships that could be construed as a potential conflict of interest.

## Footnotes

1 www.atlas-biolabs.de

2 www.ebi.ac.uk/arrayexpress

## ABBREVIATIONS

CERK1: chitin elicitor receptor kinase 1
COs: chitin oligomers
CSSP: common signaling symbiosis pathway
GSA: glucosidase SA conjugate
GSE: germinating spore exudates
GUS: beta-glucuronidase
LCO: lipo-chitooligosaccharide
LjCCD7: Lotus japonicus carotenoid cleavage dioxygenase 7
LjERF19: Lotus japonicus ethylene responsive factor 19
LjGST: Lotus japonicus glutathione-S-transferase
LjHydr: Lotus japonicus hydrolase
LjLeuc: Lotus japonicus Bark leucoagglutinin precursor
LjMATE1: Lotus japonicus multidrug and toxic compound extrusion protein
LjMTN19: Lotus japonicus medicago truncatula nodulin-like
LjPR10: Lotus japonicus pathogenesis related 10
LjVap-a: Lotus japonicus vapyrin-a
LjVap-b: Lotus japonicus vapyrin-b
LysM: lysin motif
LysM RLK: lysin motif receptor like kinase
MtERF19: Medicago truncatula ethylene responsive factor 19
MtLYK3: Medicago truncatula LysM-type receptor-like kinase 3
MtNFP: Medicago truncatula Nod factor receptor protein
Myc-LCO: mycorrhizal lipo-chitooligosaccharide
nsMyc-LCOs: non-sulphated mycorrhizallipo-chitooligosaccharide
NSP1: nodulation signaling pathway 1
NSP2: nodulation signaling pathway 2
PAMP: pathogenassociated molecular pattern
PTI: PAMP-triggered immunity
RAM: required for arbuscular mycorrhization
ROS: reactive oxygen species
SA: salicylic acid
sMyc-LCO: sulphated mycorrhizal lipo-chitooligosaccharide
